# Imaging Predictors of Silent Brain Lesions: Correlating Carotid Plaque Features on Ultrasound and CT in an Observational Study

**DOI:** 10.3390/jcm15072511

**Published:** 2026-03-25

**Authors:** Perica Mutavdzic, Tijana Kokovic, Ivan Tomic, David Matejevic, Marko Dragas, Nikola Ilic, Borivoje Lukic, Marko Miletic, Aleksandar Tomic, Igor Koncar

**Affiliations:** 1 Faculty of Medicine, University of Belgrade, 11000 Belgrade, Serbia; mutavdzic_perica@yahoo.com (P.M.); iv.tomic@yahoo.com (I.T.); markobmf@gmail.com (M.D.); fosafosa75@yahoo.com (N.I.); drboral83@gmail.com (B.L.); dr.koncar@gmail.com (I.K.); 2 Clinic for Vascular and Endovascular Surgery, Clinical Center of Serbia, 11000 Belgrade, Serbia; 3 Center for Radiology, University Clinical Center of Vojvodina, 21000 Novi Sad, Serbia; kokovic.tijana@gmail.com; 4 Center for Radiology, University Clinical Center of Serbia, 11000 Belgrade, Serbia; drmarkomiletic@gmail.com; 5 Medical Faculty, Military Medical Academy, Belgrade Defence University, 11040 Belgrade, Serbia; tomicdoc@gmail.com

**Keywords:** asymptomatic carotid stenosis, silent brain lesions, plaque vulnerability, duplex ultrasound, computed tomography angiography, lipid-rich plaque, carotid ulceration

## Abstract

**Background/Objectives:** Risk stratification in asymptomatic carotid stenosis has traditionally relied on the degree of luminal narrowing; however, plaque vulnerability may better predict cerebrovascular events. Ipsilateral silent brain lesions (SBLs) are considered surrogate markers of stroke risk. This study aimed to identify carotid plaque features on duplex ultrasound (DUS) and computed tomography angiography (CTA), as well as circulating biomarkers, associated with ipsilateral SBL in patients with clinically asymptomatic ≥70% internal carotid artery stenosis. **Methods:** This prospective observational study with cross-sectional imaging analysis included 316 clinically asymptomatic patients with ≥70% carotid stenosis treated between January 2022 and October 2024. All patients underwent cranial non-contrast CT for SBL detection, DUS plaque characterization (according to the Gray–Weale classification and plaque surface morphology), and CTA analysis, including plaque surface, composition, length, and attenuation values categorized according to Schroeder’s criteria (<50 HU lipid-rich; 51–120 HU fibrous; >120 HU calcified). Demographic, clinical, and laboratory parameters, including inflammatory biomarkers, were recorded. Multivariate logistic regression was performed to identify independent predictors of SBL. **Results:** SBL were detected in 72 patients (22.8%). On DUS, SBL were significantly associated with Gray–Weale class II plaques, heterogeneous composition, and irregular or ulcerated surfaces (all *p* < 0.001). On CTA, lipid-rich plaques (<50 HU), ulcerated surfaces, heterogeneous morphology, and lower median plaque density were significantly more frequent in the SBL group (all *p* < 0.001). In multivariate analysis, independent predictors of SBL were male sex (OR 2.2; 95% CI 1.2–5.7; *p* = 0.029), Gray–Weale class II plaques (*p* = 0.002), lipid-rich plaque morphology (OR 21.39; 95% CI 6.86–66.76; *p* < 0.001), and ulcerated plaque surface on CTA (OR 20.62; 95% CI 7.37–57.68; *p* < 0.001). **Conclusions:** Specific ultrasound and CT plaque characteristics were associated with ipsilateral silent brain lesions in patients with asymptomatic ≥70% carotid stenosis. A multiparametric imaging approach may improve risk stratification beyond stenosis severity alone.

## 1. Introduction

The identification of “vulnerable” atherosclerotic plaque plays a critical role in clinical decision-making, as such lesions represent key targets for intensive medical therapy or intervention [1]. Risk stratification for carotid stenosis has traditionally been based on the degree of luminal narrowing [2]. It has been shown that carotid plaques may remain stable for prolonged periods; however, their rupture—representing a transition to unstable or vulnerable plaques—is unpredictable and can result in clinical manifestations such as transient ischemic attack, ischemic stroke, or amaurosis fugax [3]. Therefore, increasing attention has been directed toward identifying imaging markers of plaque vulnerability that may help identify asymptomatic patients at higher risk of cerebrovascular events. The European Society for Vascular Surgery (ESVS) guidelines propose “high risk features” (HRFs) that should be considered when assessing patients with asymptomatic carotid stenosis [4]. These HRFs are heterogeneous parameters of carotid plaque, such as stenosis progression, plaque lucency, juxtaluminal black area, and intraplaque hemorrhage, obtained and quantified using various imaging modalities, including ultrasound, computerized plaque analysis, MRI, and CT. Ipsilateral silent brain lesions (SBLs) have been considered predictors of stroke; however, previous studies often used information on ischemic lesions as surrogate markers for stroke [5,6]. Plaque lucency alone is not sufficient for identifying plaque vulnerability. Although echo-lucent plaques are often associated with lipid-rich cores and increased rupture risk, plaque instability is a complex and multifactorial process [7]. Other important features include fibrous cap thickness, intraplaque hemorrhage, inflammation, positive vascular remodeling, and surface irregularities such as ulceration. Modern imaging techniques, including advanced ultrasound, computed tomography angiography, and magnetic resonance imaging, enable the assessment of these additional parameters. Therefore, the evaluation of plaque vulnerability should rely on a multiparametric approach rather than plaque lucency alone [8]. Detection of stenosis progression requires serial follow-up examinations, which are often impractical and limited by measurement variability. In addition, progression of stenosis does not necessarily reflect plaque vulnerability, potentially leading to underestimation of clinically relevant risk [9].

While magnetic resonance imaging (MRI) provides a detailed assessment of plaque composition and vulnerability, its high cost and limited accessibility raise questions about its routine use for risk evaluation. Computed tomography (CT) may offer a more economical and widely available alternative, particularly for assessing plaque morphology and calcification. Therefore, the choice of imaging modality should balance diagnostic accuracy with cost-effectiveness and availability [10]. The latest ESVS clinical practice guidelines on atherosclerotic carotid and vertebral artery disease focus primarily on imaging and anatomical criteria for management and do not include recommendations for the use of humoral biomarkers in risk assessment [4].

The aim of the study was to identify carotid plaque features on ultrasound and computed tomography, as well as circulating biomarkers, that are associated with the presence of ipsilateral silent brain lesions in patients with clinically asymptomatic ≥70% carotid stenosis.

## 2. Materials and Methods

This prospective observational study with cross-sectional imaging analysis was conducted in accordance with the STROBE (Strengthening the Reporting of Observational Studies in Epidemiology) guidelines.

The study included clinically asymptomatic patients (without previous symptoms of carotid disease—stroke, transient ischemic attack, amaurosis fugax), examined at the Clinic for Vascular and Endovascular Surgery, University Clinical Centre of Serbia, between January 2022 and October 2024. The inclusion criteria comprised asymptomatic patients with internal carotid artery stenosis greater than 70%, as determined by the initial Duplex ultrasonography according to the NASCET criteria. The study was approved by the Ethics Committee of the University Clinical Centre of Serbia and the Ethics Committee of the Faculty of Medicine, University of Belgrade (Approval No. 1322/IX-5; approved on 22 September 2022), and was conducted in accordance with the principles of the Declaration of Helsinki.

Exclusion criteria were as follows: symptoms of carotid disease (all patients were evaluated through a detailed medical history and neurological examination to confirm the absence of previous cerebrovascular symptoms including transient ischemic attack, ischemic stroke, or amaurosis fugax); carotid artery stenosis less than 70%; occlusion of the internal carotid artery; any previous hemorrhagic or ischemic stroke; non-atherosclerotic carotid disease; significant atherosclerotic changes in the aortic arch; hypercoagulable conditions (congenital or acquired thrombophilia, malignant disease, atrial fibrillation, significant heart failure and valvular disease).

Upon signing informed consent, all patients underwent a non-contrast cranial CT scan, assessed by neuroradiologists. Non-contrast cranial CT was used to detect silent brain lesions because it is widely available and routinely performed in clinical practice. However, we acknowledge that CT may be less sensitive than MRI for detecting small or subtle ischemic lesions.

Identified ischemic brain changes were classified according to Stevens’ classification: (1) normal CT findings—absence of infarct lesions; (2) discrete subcortical infarcts—well-demarcated hypodense lesions larger than 1 cm, located adjacent to healthy brain cortex in the territory supplied by the anterior and middle cerebral arteries; (3) large cortical infarcts—primarily infarcts of the cerebral cortex involving more than 50% of the territory supplied by the anterior and middle cerebral arteries; (4) small cortical infarcts—infarcts of the cerebral cortex involving less than 50% of the anterior and middle cerebral artery territories; (5) watershed infarcts/hemodynamic infarcts/hypoperfusion-related infarcts—hypodensities affecting cortical and subcortical regions at the periphery of the middle cerebral artery territory; (6) diffuse hypodense changes in the white matter—areas of decreased density, often poorly defined, especially around the periventricular regions; (7) ischemic lesions of the basal ganglia—infarcts (hypodense lesions >1 cm in diameter); (8) lacunes (hypodense lesions <1 cm in diameter) located in the striatum [11].

Demographic and clinical characteristics of the patients were recorded, with special focus on atherosclerosis risk factors, including age, body mass index (BMI), hypertension, coronary artery disease, history of myocardial infarction, percutaneous coronary intervention (PCI) or coronary artery bypass grafting (CABG), chronic obstructive pulmonary disease (COPD), diabetes mellitus, chronic kidney disease, smoking, hyperlipidemia, and peripheral arterial occlusive disease. Additionally, any prior surgical procedure involving the contralateral internal carotid artery was documented.

In all patients, laboratory parameters were collected, including total cholesterol, HDL cholesterol, LDL cholesterol, triglycerides, fibrinogen, C-reactive protein (CRP), erythrocyte sedimentation rate (ESR) and D-dimer. In addition to these conventional atherosclerosis markers, we measured several novel biomarkers: serum amyloid A (SAA), interleukin-6 (IL-6), interleukin-1β (IL-1β), matrix metalloproteinases (MMP-2, MMP-7, and MMP-9), tumor necrosis factor-alpha (TNF-α), lipoprotein-associated phospholipase A2 (LpPLA2).

Duplex ultrasound (DUS) examinations were performed by an angiologist and a vascular surgeon using a Siemens Acuson P500 machine with a linear probe (3.5–13 MHz), following manufacturer-recommended protocols for carotid artery assessment. Three key plaque characteristics were evaluated: (I) echogenicity, classified according to the Gray–Weale classification into five types: type I (uniformly echolucent), type II (predominantly echolucent), type III (predominantly echogenic), type IV (uniformly echogenic), and type V (heavily calcified with acoustic shadowing) [12]; (II) plaque surface, categorized as regular, irregular and ulcerated; and (III) plaque composition, categorized as homogeneous or heterogeneous. The degree of stenosis was expressed as a percentage based on the cross-sectional area of the residual lumen and confirmed by measuring the peak systolic velocity (PSV) and the PSV ratio between the internal and common carotid arteries. Patients were grouped according to stenosis severity: (1) 70–79%, (2) 80–89%, and (3) 90–99% stenosis. Stenosis of the contralateral internal carotid artery was also recorded and categorized as: ≤50%, 51–70%, 71–99%, or occlusion. Inter-observer agreement for ultrasound plaque morphology classification was assessed using the kappa coefficient and demonstrated good agreement between observers.

All patients underwent CT angiography (CTA) of the carotid arteries using a Toshiba Aquilion ONE 640 scanner. This scanner features 320 detector rows, and each detector can display two slices through software reconstruction, resulting in 640 slices of 0.25 mm thickness captured in a single rotation of the tube. The scanner also features a rotation speed of 0.35 s and a 72 kW generator. The topogram included the aortic arch, moving cranially to fully visualize the arteries of the Circle of Willis. Axial images were reconstructed at 0.75 mm slice thickness. The data were then processed on a workstation to generate multiplanar reconstructions (MPRs) and maximum intensity projection (MIP) images.

CTA was used to assess plaque surface morphology, composition, and quality. Plaque surface morphology was evaluated and categorized into three groups: regular, irregular, and ulcerated. Plaque composition was classified as either homogeneous or heterogeneous. CTA plaque analysis was performed independently by two experienced vascular imaging specialists with more than 10 years of experience in carotid imaging. Both observers were blinded to the presence or absence of silent brain lesions on cranial CT. Plaque quality was assessed based on its composition in relation to lipid, fibrous, or calcified components. For this purpose, attenuation measurements were obtained using an elliptical region-of-interest (ROI) cursor. Regions of interest were placed at predefined locations along the plaque (proximal, middle, and distal segments), carefully avoiding areas of heavy calcification and imaging artifacts. A total of 16 Hounsfield Unit (HU) measurements were obtained for each plaque to better capture plaque heterogeneity. The degree of stenosis for each carotid artery was categorized from 70% to 95%, and plaque length was measured and expressed in centimeters. The degree of contralateral internal carotid artery stenosis was also recorded. The sixteen HU measurements for each plaque were grouped into three categories according to Schroeder’s classification: (1) lipid-rich plaque, attenuation < 50 HU; (2) fibrous plaque, attenuation 51–120 HU; and (3) calcified plaque, attenuation > 121 HU. Based on the obtained HU values, the dominant plaque component was determined as the category with the highest frequency among the sixteen measurements.

Statistical analyses were performed using the software IBM SPSS (Statistical Package for the Social Sciences) version 25 (IBM Corp., Armonk, NY, USA). The paired t-test and one-factor analysis of variance (ANOVA) of repeated measurements were used in the paper to examine differences. A *p*-value < 0.05 was considered statistically significant. As part of the research, the analysis of agreement between researchers was examined using the kappa coefficient.

## 3. Results

During the specified period, 316 patients were divided into two groups based on the presence or absence of silent brain lesions detected on cranial CT scans: SBL group—patients with silent brain lesions (categories 2, 3, 4 and 7 according to Stevens’ classification) (72 patients, 22.8%) and non-SBL group—patients in other categories (244 patients, 77.2%). The demographic and basic clinical characteristics of these two subgroups are presented in Table 1. In both groups, male patients were more prevalent than female patients, with a statistically significantly higher proportion in the group with SBL (59.4% vs. 76.4%, *p* = 0.009). Females and patients with hypertension were statistically more represented in the asymptomatic “non-SBL” group (40.6% vs. 23.6%, *p* = 0.013) and (93.9% vs. 84.7%, *p* = 0.013), respectively.

Laboratory parameters from “non-SBL” group and the group with SBL are presented in Table 2.

A comparison of laboratory parameters was performed using the Mann–Whitney test, and the results are presented as medians and interquartile ranges (25th and 75th percentiles) for both groups in Table 3. Most laboratory parameters did not differ significantly between the two groups. However, patients with silent brain lesions had significantly higher levels of fibrinogen (3.7 (3.3–4.2) vs. 3.9 (3.5–4.4), *p* = 0.036), and TNF-α (47.06 (23.08–67.75) vs. 86.63 (68.96–119.17, *p* = 0.034) compared with patients without SBL.

The parameters assessed by Duplex ultrasonographic examination are presented in Table 4. The group with SBL had a higher percentage of patients with Gray–Weale class II plaques (4.5% vs. 16.7%, *p* < 0.001), class III plaques (31.6% vs. 45.8%, *p* < 0.001), irregular plaque surface (27.5% vs. 40.3%, *p* < 0.001), ulcerated plaque surface (4.1% vs. 15.3%, *p* < 0.001) and heterogeneous plaque composition (33.6% vs. 56.9%, *p* = 0.001). On the other hand, the non-SBL patient group had a higher percentage of patients with Gray–Weale class IV carotid plaques (59.4% vs. 37.5%, *p* < 0.001) and regular plaque surface (68.4% vs. 44.4%, *p* < 0.001). No statistically significant differences were recorded in the degrees of stenosis of the examined carotid arteries or contralateral ICA in both groups.

Parameters diagnosed by computed tomography of carotid plaque are shown in Table 5. The SBL-group had a higher percentage of patients with: mean plaque density between ≤50 HU (0.8% vs. 2.8%, *p* = 0.019), mean plaque density between 50 and 120 HU (7.4% vs. 18.1%, *p* = 0.010), predominantly lipid-rich plaque morphology (4.1% vs. 38.9%, *p* < 0.001), ulcerated plaque surface (5.3% vs. 36.1%, *p* < 0.001), and heterogeneous plaque composition (25.8% vs. 56.9%, *p* < 0.001). On the other hand, the non-SBL group had a higher percentage of patients with mean plaque density over 120 HU (91.8% vs. 79.2%, *p* < 0.001), predominantly calcified plaque morphology (78.7% vs. 40.3%, *p* < 0.001), smooth plaque surface (63.5% vs. 23.6%, *p* < 0.001), homogeneous plaque composition (72.4% vs. 43.1%, *p* < 0.001) and higher median plaque density measured in HU (614 [274–911] vs. 240 [136–513], *p* < 0.001).

In the multivariate regression model, the following independent predictors were identified as influencing the occurrence of SBL in patients with asymptomatic carotid artery disease (Table 6):

- Male patients have a 2.21 times higher likelihood of developing SBL compared to females (OR = 2.2, 95% CI: 1.2–5.7, *p* = 0.029)
- Gray–Weale class II diagnosed by DUS (OR = 1.65, 95% CI: 0.97–4.32, *p* = 0.002)
- Lipid plaque morphology (CT) (< 50 HU) (OR = 21.39, 95% CI: 6.86–66.76, *p* < 0.001)
- Ulcerated plaque surface (on CT) (OR = 20.62, 95% CI: 7.37–57.68, *p* < 0.001)

In summary, high-risk patients with asymptomatic carotid artery disease are best identified through a multifactorial approach that integrates plaque vulnerability, stenosis progression, and cerebrovascular reserve. This strategy allows for individualized risk assessment and may guide therapeutic decision-making to prevent both silent brain lesions and future ischemic strokes, highlighting the importance of advanced imaging and functional testing in routine clinical practice.

## 4. Discussion

The present prospective study evaluated the occurrence of SBL in 316 clinically asymptomatic patients with carotid artery stenosis and analyzed carotid plaque characteristics using CT and duplex ultrasound. The results demonstrate that several plaque features detected by ultrasound or CT are independently associated with the presence of SBL, suggesting that plaque morphology and composition may play an important role in cerebrovascular risk stratification in patients with asymptomatic carotid artery disease.

Optimal clinical management of extracranial carotid stenosis hinges on two fundamental questions: (1) what is the risk of stroke associated with the identified carotid lesion, and (2) what is the procedural risk of intervention in an individual patient? Although both questions involve numerous nuances and patient-specific factors, careful assessment of these domains is essential for selecting the most appropriate treatment strategy. In the Asymptomatic Carotid Stenosis and Risk of Stroke (ACSRS) study, the presence of silent embolic infarcts in the ipsilateral cerebral hemisphere in patients with moderate or severe asymptomatic carotid artery stenosis was associated with an increased risk of ipsilateral neurologic events and stroke compared with patients without such lesions [13]. On this basis, SBLs may be considered surrogate markers of future stroke risk, and analyses of predictors of SBLs may therefore provide valuable insight into factors associated with cerebrovascular events [4].

In the present study, the incidence of SBLs detected by brain CT in patients with asymptomatic carotid stenosis was 22.8%. This finding is consistent with previously reported rates ranging from approximately 10% to 24%. In the Asymptomatic Carotid Emboli Study (ACES), baseline SBLs were detected in 15% of patients, with 22% classified as small and 72% as deep infarcts [14,15,16]. Similarly, in our cohort, the majority of lesions were located in subcortical regions and the basal ganglia. With the increasing availability and more widespread use of computed tomography in clinical practice, silent brain lesions are being detected more frequently. Their presence may reflect prior unrecognized cerebrovascular ischemic events or may be associated with progressive brain atrophy, representing cumulative neurological damage [17,18].

Beyond their association with stroke risk, SBLs have also been linked to a significantly increased risk of mood disturbances, gait impairment, cognitive decline, and dementia [19]. Consequently, they are increasingly regarded as markers of “brain frailty.” In addition to carotid stenosis, several factors have been associated with the occurrence of SBLs, including advanced age and cardiovascular risk factors such as hypertension, atrial fibrillation, and patent foramen ovale. In the present study, patients with atrial fibrillation, patent foramen ovale, significant valvular heart disease, heart failure, ventricular aneurysms, or other conditions associated with potential cardiac embolic sources were excluded. Patients with non-atherosclerotic carotid disease, significant atherosclerotic changes in the aortic arch, or hypercoagulable conditions were also excluded in order to minimize potential confounding sources of cerebral embolization.

Carotid atherosclerosis may contribute to the development of SBLs through several mechanisms. Atherosclerotic plaques may serve as a source of microembolization, or they may induce cerebral ischemia in the presence of flow-limiting stenosis, ultimately leading to chronic hypoperfusion and brain atrophy [20,21]. Several mechanisms have been proposed, including local vascular degeneration leading to hypoperfusion, acute ischemia caused by distal embolization originating from the heart or large arteries, and chronically reduced cerebral perfusion [22,23]. Repeated showers of small microemboli, composed of platelet aggregates or cholesterol crystals, may be released from the surface of unstable carotid plaques [24]. Such infarcts have therefore been suggested to represent markers of active carotid plaque ulceration, based on correlations between postoperative examinations and CT findings in the same patients [25].

Recent ESVS guidelines emphasize the importance of plaque imaging characteristics for risk stratification in patients with asymptomatic carotid stenosis. The combination of anatomical, morphological, and functional parameters may provide a comprehensive framework for identifying patients who could benefit from intensified surveillance, optimized medical therapy, or early carotid revascularization [3,13,14]. However, many of the proposed markers have important practical limitations. Certain imaging techniques may not be widely available, while others require specialized software or longitudinal follow-up. The present study therefore focused on imaging parameters that are widely available in routine clinical practice, including duplex ultrasonography and CT-based plaque analysis.

In our cohort, plaques with low attenuation (<50 HU) on CT and ulcerated plaque surfaces were associated with significantly higher odds of SBLs. Additionally, Gray–Weale class II plaques identified by duplex ultrasound were independently associated with SBL occurrence. These findings support the concept that plaque composition and morphology are key determinants of cerebrovascular risk. Vulnerable plaques characterized by lipid-rich necrotic cores, low echogenicity, or ulcerated surfaces are more prone to rupture and embolization, thereby increasing the risk of both silent and symptomatic cerebrovascular events. Multimodal imaging approaches combining different modalities may therefore allow clinicians to identify high-risk carotid plaques beyond the degree of stenosis alone. For example, plaques that appear echolucent on ultrasound or demonstrate low attenuation on CT are more likely to rupture and act as embolic sources [24,25]. A multiparametric assessment integrating plaque morphology, composition, and hemodynamic characteristics may therefore improve patient selection for intervention and refine current risk stratification strategies.

Carotid atherosclerosis may also be regarded as a marker of increased ischemic vulnerability of the brain, emphasizing the need for aggressive management of cardiovascular risk factors. This includes optimal medical therapy with antiplatelet agents such as aspirin and lipid-lowering therapy with statins, which have been shown to slow the progression of atherosclerotic disease and reduce the risk of cerebrovascular events [26]. Although many patients with asymptomatic carotid stenosis remain stroke-free for prolonged periods, a subset of individuals carries a substantially higher risk due to a combination of anatomical, hemodynamic, and biological factors [3,22]. High-risk patients are generally characterized by rapid progression of stenosis, the presence of vulnerable plaque morphology, and impaired cerebrovascular compensatory mechanisms [23,25,27].

Hemodynamic assessment may represent another important aspect of risk stratification. Impaired cerebrovascular reactivity, measured using functional imaging techniques or transcranial Doppler methods, has been shown to predict stroke risk independently of stenosis severity. Patients with reduced cerebrovascular reserve are less able to compensate for decreases in cerebral perfusion and may therefore be more susceptible to ischemic injury in the presence of plaque instability or transient embolic events [27]. Rather than focusing primarily on hemodynamic parameters, the present study was designed to explore the relationship between carotid plaque morphology and ischemic cerebral injury, specifically the occurrence of silent brain lesions.

The relatively high odds ratios observed for lipid-rich plaques and ulcerated plaque surfaces likely reflect the strong biological association between plaque vulnerability and embolic potential. Nevertheless, these estimates should be interpreted cautiously, given the observational design of the study.

### Limitations

Several limitations of the present study should be acknowledged. First, although the study was conducted prospectively, the relatively low event rate of SBL may limit the statistical power of the analysis and the generalizability of the findings beyond the studied cohort. In addition, the study population consisted exclusively of patients with ≥70% carotid stenosis. This threshold was selected because it corresponds to current guideline recommendations for considering carotid revascularization in asymptomatic patients; however, this inclusion criterion may limit the applicability of the results to individuals with lower degrees of stenosis.

Second, CT was used for the detection of silent brain lesions due to its wide availability and routine clinical use. However, MRI is more sensitive for identifying small or subclinical cerebral infarctions, and the true prevalence of SBL may therefore have been underestimated.

Third, although duplex ultrasound and multidetector computed tomography (MDCT) are widely available and clinically practical imaging modalities, they have inherent limitations in sensitivity and specificity for detailed plaque characterization. Certain plaque components, such as lipid-rich necrotic cores or intraplaque hemorrhage, may be more accurately detected using higher-resolution imaging techniques or histopathological analysis. Ultrasound-based plaque assessment is also operator dependent and may be subject to measurement variability.

Fourth, contrast-enhanced ultrasound, which can provide additional information regarding plaque neovascularization and vulnerability, was not available at our institution during the study period and therefore could not be included in the imaging protocol.

Fifth, potential confounding from cerebral small vessel disease and other vascular risk factors cannot be completely excluded and may have contributed to the occurrence of silent brain lesions independently of carotid plaque morphology.

Finally, the study design did not include long-term follow-up, preventing evaluation of the prognostic significance of the identified plaque characteristics beyond the initial detection of SBL. Apart from the small size of these infarcts, other factors may contribute to their clinically silent presentation. Cerebral microembolization occurring during sleep may remain unnoticed, and transient neurological symptoms may not always be recognized or reported by patients. Furthermore, no consistent localization of lesions to so-called “silent” brain regions was observed in this study, an observation that has also been reported in previous investigations [28].

## 5. Conclusions

In patients with clinically asymptomatic ≥70% carotid artery stenosis, specific carotid plaque characteristics assessed by duplex ultrasound and computed tomography are independently associated with the presence of silent brain lesions. In particular, lipid-rich plaque morphology and ulcerated plaque surfaces on CT, as well as echolucent plaques on ultrasound, emerged as strong predictors of subclinical cerebral ischemic injury.

These findings support the concept that plaque vulnerability, rather than stenosis severity alone, plays a central role in cerebrovascular risk. A multimodal imaging approach integrating plaque morphology and composition may therefore improve risk stratification and help identify a subgroup of asymptomatic patients at higher risk who may benefit from closer surveillance or earlier intervention.

However, given the cross-sectional design of the study, further longitudinal investigations are required to determine the prognostic significance of these imaging features for future cerebrovascular events and to refine their role in clinical decision-making.

## Figures and Tables

**Table 1:** Basic demographic and clinical characteristics of the population.

Variables	Non-SBL Group	SBL Group	*p*-Value
	**n = 244**	**n = 72**	
**Demographics**			
Age (years)	70 (65–74)	69 (66–75)	0.902
Male sex	145 (59.4)	55 (76.4)	**0.009**
Female sex	99 (40.6)	17 (23.6)	**0.013**
BMI	25.8 (23.9–27.8)	25.3 (23.4–28.2)	0.539
**Risk factors**			
Current smoker	74 (30.3)	21 (29.2)	0.304
Former smoker	56 (23.0)	13 (18.1)	0.590
Hypertension	229 (93.9)	61 (84.7)	**0.013**
Hyperlipidemia	104 (42.6)	32 (44.4)	0.784
Diabetes mellitus	95 (38.9)	24 (33.3)	0.389
Coronary artery disease	61 (25.0)	16 (22.2)	0.629
Previous MI	34 (13.9)	9 (12.5)	0.755
Previous PCI/CABG	35 (14.3)	12 (16.7)	0.627
COPD	28 (11.5)	5 (6.9)	0.380
Chronic kidney disease	27 (11.1)	8 (11.1)	0.991
Peripheral artery disease	53 (21.7)	11 (15.3)	0.232
Previous ICA surgery	45 (18.4)	9 (12.5)	0.239

Data are presented as n (%), or as mean and median/interquartile range. BMI—body mass index; COPD—chronic obstructive pulmonary disease; MI—myocardial infarction; PCI—percutaneous coronary intervention; CABG—coronary artery bypass grafting; ICA—internal carotid artery.

**Table 2:** Examined laboratory parameters.

Variables	Non-SBL Group	SBL Group	*p*-Value
	**n = 244**	**n = 72**	
			
Cholesterol > 5.2 mmol/L	92 (37.8)	36 (48.1)	0.121
HDL < 1.2 mmol/L	94 (38.7)	34 (47.2)	0.195
LDL > 3.4 mmol/L	54 (22.1)	18 (25.0)	0.610
Triglycerides > 1.7 mmol/L	76 (31.1)	26 (36.1)	0.429
CRP > 8 mg/L	40 (16.4)	7 (9.7)	0.162
ESR > 20 mm/h	116 (47.5)	29 (40.3)	0.277
D-dimer > 0.5 mg/L	112 (45.9)	40 (55.6)	0.150
Fibrinogen > 4.0 g/L	97 (39.8)	25 (34.7)	0.441

Data are presented as n (%)**.** HDL—high-density lipoprotein cholesterol; LDL—low-density lipoprotein cholesterol; CRP—C-reactive protein; ESR—erythrocyte sedimentation rate.

**Table 3:** Laboratory parameters comparison.

Variables	Non-SBL Group	SBL Group	*p*-Value
	**n = 244**	**n = 72**	
			
Cholesterol (mmol/L)	4.51 (3.65–5.17)	4.54 (3.89–5.30)	0.409
HDL (mmol/L)	1.26 (1.08–1.51)	1.22 (1.04–1.42)	0.204
LDL (mmol/L)	2.42 (1.90–3.19)	2.49 (1.97–3.31)	0.678
Triglycerides (mmol/L)	1.38 (1.05–1.87)	1.48 (1.07–1.90)	0.410
CRP (mg/L)	2.50 (1.00–6.15)	2.60 (1.30–4.50)	0.893
ESR (mm/h)	20 (14–30)	19 (10–29)	0.122
D-dimer (mg/L)	0.50 (0.36–0.92)	0.55 (0.39–0.93)	0.277
Fibrinogen (g/L)	3.7 (3.3–4.2)	3.9 (3.5–4.4)	**0.036**
IL-6 (pg/mL)	2.55 (1.88–15.78)	14.88 (7.61–16.77)	0.303
IL-1β (pg/mL)	51.05 (23.93–75.10)	30.56 (8.75–69.68)	0.272
MMP-2 (ng/mL)	244.04 (205.88–319.66)	254.32 (210.53–326.35)	0.818
MMP-7 (ng/mL)	5.37 (3.24–9.39)	6.45 (2.54–10.84)	1.000
MMP-9 (ng/mL)	319.66 (224.17–418.93)	308 (217.01–353.44)	0.869
TNF-α (pg/mL)	47.06 (23.08–67.75)	86.63 (68.96–119.17)	**0.034**
LpPLA2 (ng/mL)	222.98 (128.51–276.44)	199.45 (81.41–271.83)	0.669
SAA (mg/L)	3.20 (2.48–5.08)	2.34 (2.06–2.86)	0.128

Data are presented as n (%), or as mean and median/interquartile range. HDL—high-density lipoprotein cholesterol; LDL—low-density lipoprotein cholesterol; CRP—C-reactive protein; ESR—erythrocyte sedimentation rate; SAA—serum amyloid A, Il-6—interleukin-6; Il-1β—interleukin-1β, MMP-2—matrix metalloproteinase 2; MMP-7—matrix metalloproteinase 7; MMP-9—matrix metalloproteinase 9; TNF-α—tumor necrosis factor-alpha; LpPLA2—lipoprotein-associated phospholipase A2.

**Table 4:** Duplex ultrasonographic characteristics.

Variables	Non-SBL Group	SBL Group	*p*-Value
	n = 244	n = 72	
			
Gray–Weale Class II	11 (4.5)	12 (16.7)	**<0.001**
Gray–Weale Class III	77 (31.6)	33 (45.8)	**<0.001**
Gray–Weale Class IV	145 (59.4)	27 (37.5)	**<0.001**
Gray–Weale Class V	10 (4.1)	0 (0)	
Plaque surface			
- regular	167 (68,4)	32 (44.4)	**<0.001**
- irregular	67 (27.5)	29 (40.3)	**<0.001**
- ulcerated	10 (4.1)	11 (15.3)	**<0.001**
Heterogeneous plaque composition	82 (33.6)	41 (56.9)	**0.001**
Degree of stenosis			
- 70–79%	60 (24.6)	13 (18.1)	0.451
- 80–89%	105 (43)	35 (48.6)	0.476
- 90–99%	79 (32.4)	24 (33.3)	0.488
PSV (peak systolic velocity)	285 (254–361)	284 (266–380)	0.211
PSV ratio	5.6 (4.0–7.6)	5.69 (4.45–7.35)	0.700
Contralateral stenosis			
- to 50%	112 (45.9)	39 (54.2)	0.595
- 51–70%	45 (18.4)	10 (13.9)	0.607
- 71–99%	64 (26.2)	15 (20.8)	0.628
- occlusion	23 (9.4)	8 (11.1)	0.540

**Table 5:** CT-angiographic characteristics of the two groups.

Variables	Non-SBL Group	SBL Group	*p*-Value
	**n = 244**	**n = 72**	
Contralateral stenosis			
- to 50%	114 (46.7)	40 (55.6)	0.449
- 50–69%	43 (17.6)	10 (13.9)	0.382
- 70–99%	64 (26.2)	14 (19.4)	0.518
- occlusion	23 (9.4)	8 (11.1)	0.456
Mean plaque density (HU *)			
- to 50 HU	2 (0.8)	2 (2.8)	**0.019**
- 50–120 HU	18 (7.4)	13 (18.1)	**0.010**
- over 120 HU	224 (91.8)	57 (79.2)	**0.004**
Median plaque density (HU)	614 (274–911)	240 (136–513)	**<0.001**
Plaque morphology			
- lipid-rich	10 (4.1)	28 (38.9)	**<0.001**
- fibrous	42 (17.2)	15 (20.8)	0.464
- calcified	192 (78.7)	29 (40.3)	**<0.001**
Plaque surface			
- regular	155 (63.5)	17 (23.6)	**<0.001**
- irregular	76 (31.1)	29 (40.3)	0.134
- ulcerated	13 (5.3)	26 (36.1)	**<0.001**
Plaque composition	104 (32.9)	53 (55.2)	**<0.001**
- homogeneous	181 (72.4)	31 (43.1)	**<0.001**
- heterogeneous	63 (25.8)	41 (56.9)	**<0.001**
Degree of stenosis			
- 70–79%	65 (26.6)	12 (16.7)	0.166
- 80–89%	80 (32.8)	30 (41.7)	0.161
- 90–99%	99 (40.6)	30 (41.7)	0.311
Median degree of stenosis	85 (75–90)	85 (80–90)	0.121
Plaque length (cm)	2.0 (1.5–2.4)	2.1 (1.7–2.4)	0.435

* HU—Hounsfield Units.

**Table 6:** Uni- and multivariate logistic regression analysis of risk factors associated with the occurrence of SBL in patients with asymptomatic carotid artery disease.

Variables	Univariate Analysis			Multivariate Analysis		
	OR	95%CI	*p* Value	OR	95%CI	*p* Value
male sex	2.20	1.21–4.03	**0.001**	2.21	1.2–5.7	**0.029**
intraplaque hemorrhage	7.98	0.81–78.43	0.075			
irregular plaque surface (DUS)	2.26	1.27–4.02	**0.006**	1.39	0.68–2.82	0.634
ulcerated plaque surface (DUS)	5.74	2.25–14.64	**<0.001**	2.05	0.52–8.11	0.305
heterogeneous plaque (DUS)	2.61	1.53–4.47	**<0.001**	1.55	0.81–2.97	0.189
Gray–Weale class II	0.42	0.29–0.63	**<0.001**	1.65	0.97–4.32	**0.002**
↓ mean plaque density (HU)	0.40	0.21–0.76	**0.005**			
lipid plaque morphology (CT)	18.54	8.16–42.13	**<0.001**	21.39	6.86–66.76	**<0.001**
fibrous plaque morphology (CT)	2.37	1.17–4.78	**0.017**	1.77	0.71–4.45	0.221
heterogeneous plaque (CT)	3.8	2.20–6.57	**<0.001**	1.21	0.56–2.60	0.627
irregular plaque surface (CT)	3.47	1.80–6.72	**<0.001**	3.52	1.54–8.05	0.003
ulcerated plaque surface (CT)	18.24	7.93–41.95	**<0.001**	20.62	7.37–57.68	**<0.001**
TNF-α	0.97	0.93–1.00	**0.077**			
fibrinogen	0.74	0.52–1.06	**0.102**			

The data are presented as odds ratios with a 95% confidence interval (OR 95% CI).

## Data Availability

The original contributions presented in this study are included in the article. Further inquiries can be directed to the corresponding author.
